# Supplementary material for: Assessing the performance of a commercial multisensory sleep tracker
Source: PLoS One. 2020 Dec 11;15(12):e0243214. doi: 10.1371/journal.pone.0243214 (PMC7732119; doi:10.1371/journal.pone.0243214)
Supplement: Attachment — Submitted filename: Response_to_Reviewers.pdf [file pone.0243214.s001.pdf]

August 2020

Dear Academic Editor and Reviewers,

We thank you for your generous comments on our manuscript. We hereby send the rebuttal letter, and to the best of our ability, we have responded to all of your comments.

Thank you for the recommendation of protocols.io. We do not believe this will be of interest since our protocol was formulated in Danish.

Journal requirements

1. *Please ensure that your manuscript meets PLOS ONE's style requirements, including those for file naming. The PLOS ONE style templates can be found at https://journals.plos.org/plosone/s/file?id=wjVg/PLOSOne_formatting_sample_main_body.pdf and https://journals.plos.org/plosone/s/file?id=ba62/PLOSOne_formatting_sample_title_authors_affiliations.pdf*

We ensured that our manuscript meets PLOS ONE's style and file naming requirements.

2. *Thank you for including your ethics statement: 'The trial protocol was approved by the Regional Health Research Ethics Committee (SJ-780).'*

a. *Please amend your current ethics statement to include the full name of the ethics committee/institutional review board(s) that approved your specific study.*

b. *Once you have amended this/these statement(s) in the Methods section of the manuscript, please add the same text to the "Ethics Statement" field of the submission form (via "Edit Submission").*

Both of these points have been amended. We added: "*The trial protocol was approved by the Ethics Committee of Region Zealand (protocol number: SJ-780)*".

For additional information about PLOS ONE ethical requirements for human subjects research, please refer to http://journals.plos.org/plosone/s/submission-guidelines#loc-human-subjects-research

3. *Thank you for including your competing interests statement; "The study was based on Garmin Vivosmart 4 sleep tracking because of its multisensory technology and user-friendly design. Garmin kindly borrowed us Garmin watches to perform the experiment. This had no influence on the study design, data collection or analysis, decision to publish, or preparation of the manuscript."*

Please confirm that this does not alter your adherence to all PLOS ONE policies on sharing data and materials, by including the following statement: "This does not alter our adherence to PLOS ONE policies on sharing data and materials." (as detailed online in our guide for authors http://journals.plos.org/plosone/s/competing-interests). If there are restrictions on sharing of data and/or materials, please state these. Please note that we cannot proceed with consideration of your article until this information has been declared. Please include your updated Competing Interests statement in your cover letter; we will change the online submission form on your behalf.

We have now confirmed our adherence to PLOS ONE policies on sharing data and materials under 'Conflict of interests' by writing: "*We thank Garmin® who kindly borrowed us GV4 watches to perform our experiment. This had no influence on our study design, data analysis, results or conclusions and does not alter our adherence to the PLOS ONE policies on sharing data and materials. According to Danish law, data cannot be shared publicly. However, data are available upon request from the Zealand University Hospital, Department of Neurology, Center of Neurophysiology for researchers who meet the criteria for access to confidential data*". We also confirmed our adherence in our cover letter.

Please know it is PLOS ONE policy for corresponding authors to declare, on behalf of all authors, all potential competing interests for the purposes of transparency. PLOS defines a competing interest as anything that interferes with, or could reasonably be perceived as interfering with, the full and objective presentation, peer review, editorial decision-making, or publication of research or non-research articles submitted to one of the journals. Competing interests can be financial or non-financial, professional, or personal. Competing interests can arise in relationship to an organization or another person. Please follow this link to our website for more details on competing interests:http://journals.plos.org/plosone/s/competing-interests

Competing interests have already been declared in the manuscript and at submission. The authors have no further competing interests to declare.

Reveiwer #1

This is a validation study for a commercial sleep tracker from Garmin. The study was carried out in parallel with polysomnography, which is the reference standard for sleep recording. A small number of subjects was investigated: 18 healthy persons. One person was recorded for 23 nights, but these were not with polysomnography. The results show that the sleep tracker is good for sleep onset and end of sleep, but not good for sleep stages. This appears to be similar to actigraphy which has not been tested here.

As written in the 'Polysomnography' section, I assume that this is not a AASM type I study with supervised polysomnography, but a AASM type II study with polysomnography at home. If this is the case, please state this clearly, because this setting is different to most

settings. In type II studies, there is no video recording or no monitoring from an experienced sleep technician. This needs to be noted.

Thank you for this relevant comment. Your assumption is correct, this was an AASM type II study, and we have now provided this information under 'Polysomnography': "*Overnight home PSG (AASM Type II study) was obtained by use of TrackIT™ Mk3 Sleep Click-on 2 (Lifelines, Neuro). Sleep was unattended without monitoring by a technician or by video*".

For the smartwatch, you say that it considers not only actigraphy, but PPG as well. But how is this done? Since the results show, that these are comparable to simple actigraphy, may be PPG is not really evaluated? If we consider the WatchPAT device from Itamar as a smartwatch as well, then that device does a better job in evaluationg the PPG signal. Please compare critically.

This is an interesting observation that we also addressed in the 'Discussion'. Garmin has only disclosed to use movement and PPG signals to evaluate sleep, thus, we do not know how Garmin applies PPG data in its algorithm to assess sleep. We have simply assumed that PPG signals are applied since this was claimed by Garmin. In accordance with this, we added the following sentence in the 'Discussion': "*However, the beneficial role of PPG signals in accelerometer-based sleep tracking remains unilluminated, since we do not know how these signals are processed and applied*".

I am especially interest how the Garmin evaluates REM sleep. That it evaluates REM sleep is only mentioned in brackets in the methods and details are only given when one reads the results. I think this capability of Garmin to detect REM sleep needs to be described in the methods section.

Due to the proprietary nature of sleep algorithms used by Garmin, unfortunately we cannot elaborate exactly how Garmin detects REM sleep. Sparse information is publicly available about how sleep stages are detected.

I think it is very good to check for 23 consecutive nights and it is very good to have two devices simultaneously.

As you performed an epoch by epoch comparison, it is of interest, how you arranged for a good synchronization between the polysomnography and the Garmin device. How much was the synchronization error then, less than a second?

Both Garmin and PSG were manually synchronized using the internet clock time before each PSG study. Since Garmin's sampling frequency is 1 min, we considered an online time agreement as sufficient. This consideration has been addressed under 'Inter-device reliability' by adding: "*Both GV4 and PSG were manually synchronized using the internet clock time before each PSG study*".

The critical summary is appreciated.

Reviewer #2

Authors aimed at evaluating the performance of GV4 against PSG in measuring several sleep parameters in a small sample of 18 "healthy" adults in their home environment. I agree with the authors that the validation of such devices is an important step for adoption. The manuscript is well written.

Comments:

Please consider the guidelines outlined by Depner at al., 2020 - Wearable Technologies for Developing Sleep and Circadian Biomarkers A Summary of Workshop Discussions

Thank you for introducing this relevant paper. We read this with great interest and have incorporated it in our revised manuscript.

I would use the term "assessing the performance" instead of "validating".

Thank you for your input. We have now altered the title to "*Assessing the performance of a commercial multisensory sleep tracker*".

"without sleep disorder". How sleep disorders were evaluated? Self-report?

Yes, sleep disorders were evaluated by self-report. We added "*without self-reported sleep disorders*" under 'Abstract' and 'Methods'.

Not sure about having the intra-device reliability as an aim, which has been evaluated on a single participant. What is the generalizability of such results? You may consider this as "exploratory"

In our intra-device reliability study, we aimed to generalize our results between Garmin devices. This was in order to provide indications about the consistency between Garmin devices' outputs under identical exposures. We considered this as a prerequisite for validation of GV4 against gold standard.

Correlation analysis and interpretation of correlation outputs is misleading. Please remove it.

Thank you for this relevant comment. We are aware of the limitations related to the use of Pearson's correlations. We have now removed it and replaced it with intraclass correlations (Table 3) which has been suggested as a more appropriate measure because it both reflects correlation and agreement.

"Our results suggest inaccurate sleep stage detection by GV4, but sleep onset and sleep end were accurately detected with few outliers." The absence of a significant bias between PSG and device does not specifically inform about accuracy/inaccuracy.

This is true and has been rephrased: "*Our results suggest inaccurate sleep stage detection by GV4, but sleep onset and sleep end were highly correlated with and differed insignificantly from PSG measures*".

I would not consider low- vs high-frequency HRV as sympathetic vs vagal influence. I would quickly describe the main autonomic changes occurring across stages of sleep.

Thank you for the comment. This has been elaborated in the 'Introduction': "*When falling to sleep, parasympathetic influence gradually increases through non rapid eye movement sleep (NREM) while sympathetic influence decreases. This results in reduced cardiac activity. When progressing into rapid eye movement sleep (REM), and specifically into phasic REM, sympathetic influence increases together with cardiac activity. Optical photoplethysmography (PPG) is a mechanism that detects changes in blood volume and thereby measures heart rate (HR) and HR variability (HRV) by illuminating the skin with subsequent registration of changes in the reflected light. HRV frequencies can help indicate shifts between sleep stages*".

It is unclear how the lights-off and lights-on were identified. Participant were asked to press an event-marker button when going to bed. How the authors determined the next morning wake time?

Thank you for the relevant comment. This has been elaborated under 'Materials', 'Polysomnography': "*Participants were asked to press an event marker button when going to bed (identified as "lights off")*". The true next morning wake time was determined by PSG scored by MHL which was also elaborated under 'Data analysis', 'Inter-device reliability': "*Both GV4 and PSG were manually synchronized using the internet clock time before each PSG study. GV4 and PSG data were compared from "lights off", registered by the participant, until the first wake epoch registered by both GV4 and PSG (scored by MHL)*".

What was the rate of concordance between the 2 scorers (MHL and TWK)?

We have no measure of concordance between MHL and TWK. TWK only revised the PSG recordings after MHL had scored them.

Please provide the data collection time windows (e.g., from Aug 2018 to ...)

This has been added under 'Materials', 'Garmin Vivosmart 4': "*PSG and GV4 data were collected from December 2019 until March 2020*".

Did the authors control for normality of data distribution in the analyses?

To check for normality qq-plots were performed for all measures on both devices. We have mentioned this in under 'Data analysis': "*GV4 and PSG data were checked for normality*". There is generally close to normal distribution except for one plot (PSG SOL). Regarding this plot, the non-normal distribution of SOL has already been reported in the literature when focusing on multiple sleep latency (Sleep, 20(1):34-39 © 1997 American Sleep Disorders Association and Sleep Research Society How Should the Multiple Sleep Latency Test Be Analyzed?).

The SD of WASO for GV4 is much lower than the SD of WASO for PSG. Please comment on that.

This has been addressed in the 'Discussion': "*WASO measured by PSG had a notably larger sd than WASO measured by GV4 (Table 3). This underlines a general tendency with underestimations of WASO across participants*".

Please provide BA plots for all the sleep parameters analyzed. In the BA plots please use the PSG values on the x axis, and plot the bias or the proportional bias

BA plots for all analyzed sleep parameters are now provided in Fig. 1A-L.

EBE should be performed on an individuals' level. Sensitivity and specificity should be calculated for each participant (night) separately.

EBE analysis and calculations of sensitivity and specificity were originally performed on an individual's level. To address this comment, we elaborated the description of the calculation of these values under 'Methods': "… *were calculated as mean values from the EBE analyses performed in each participant. Those values were also calculated for each type of alignment*". We furthermore added standard deviations for sensitivity, specificity, accuracy, observed agreement and kappa values to our 'Results': "*Based on the confusion matrices, sensitivity was calculated to 0.98 ± 0.03, specificity to 0.30 ± 0.17, accuracy to 0.90 ± 0.06, observed agreement to 0.48 ± 0.10, and Cohen's kappa to 0.20 ± 0.11 for all sleep stages and 0.33 ± 0.18 for sleep/wake*".

Sensitivity and specificity should be provided for all sleep stages (separately).

These values are now provided in 'Results': *Sensitivity for light, deep, and REM sleep were calculated to 0.60 ± 0.19, 0.45 ± 0.26, and 0.34 ± 0.26, respectively. Specificity for light, deep and REM sleep were calculated to 0.49 ± 0.13, 0.83 ± 0.20, and 0.88 ± 0.08, respectively*".

Can you please clarify this statement? "The high agreement in HR and movement detection between devices indicates differences in software rather than hardware"?

Because intra-device agreement was lower than expected, we wanted to identify potential differences in the detection of movement and HR data. After extraction and observation of these, we realized that both sets of data were almost identical. Thus, we found it plausible that the relatively low agreement between devices was not due to differences in sensors (hardware) but rather to differences in data processing (software). This has been elaborated in our 'Discussion': "*The relatively low intra-device agreement may be due to differences in software (data processing) rather than hardware (sensors) since high agreement was found between devices in the detection of both HR and movement*".

The advantages of incorporating HR and HRV indices by these devices should be due to the sleep stage dependent shifting in autonomic control. Not sure what authors imply with this statement "HRV is influenced by several other conditions than sleep for instance stress level".

We wrote it to emphasize a potential limitation in the use of HR and HRV in sleep evaluation as we, in that section, just emphasized a known limitation in the use of accelerometry (motionless wake). We want motionless wake to be in focus. We realized this sentence was confusing and removed it.

I would avoid any interpretation about GV4 performance due to the limited generalizability of the results (limitation of the sample used) and the lack of standard in evaluating what could be considered acceptable or not.

Thank you for this very relevant comment. This is true and was further addressed. We have now included this important limitation in our conclusion: "*In summary, our results confirmed that GV4 is not able to reliably describe sleep architecture but may accurately detect changes in sleep onset, sleep end, and TST though generalizations are difficult due to our sample limitations. Thus, our findings should be confirmed by further studies"*. We have based our interpretations on interpretations made in similar validations studies.

Please check refs. 4, 6, 20 for accuracy.

These references have been edited for proper presentation.

We are looking forward to your respond.

On behalf of all the authors,

Best regards,
Nanna J. Mouritzen, Medical student, Corresponding author
