# Supplementary material for: Assessing the performance of a commercial multisensory sleep tracker
Source: PLoS One. 2020 Dec 11;15(12):e0243214. doi: 10.1371/journal.pone.0243214 (PMC7732119; doi:10.1371/journal.pone.0243214)
Supplement: Attachment — Submitted filename: Response_to_Reviewers_V1.2.docx [file pone.0243214.s002.docx]

Dear Academic Editor and Reviewer #2,

We thank you for your additional comments on our manuscript. We hereby send the rebuttal letter, and to the best of our ability, we have responded to your latest comments.

**Reviewer #2**

*The authors did a nice job in reviewing the paper and replying to the Reviewers’ concerns!
Few additional comments.*

*I would avoid statements like “Our results suggest inaccurate sleep stage detection by GV4”, etc. There is no standard on what would be considered accurate/inaccurate, and the picture is extremely complex (there are biases, proportional biases, clinical significance other than statistical significance to consider, etc.). Thus, I believe that statements like that give the readers false interpretation of the device performance.*

This is a very relevant point that we agree with. We excluded the use of ‘inaccurate’ and rephrased the sentence: *“Our results suggest that GV4 is not able to reliably describe sleep architecture but may allow for detection of changes in sleep onset, sleep end, and TST (ICC* $\text{≥}$ *0.825) in longitudinally followed groups. Still, generalizations are difficult due to our sample limitations”.*

*In the intro, you may want to mention that PPG-derived HR and HRV has been “validated” against ECG-derived HR and HRV.*

Thank you very much for this comment. We have focused on the reproducibility of HR estimates between two identical Garmin devices for two successive nights. We actually did not directly compare PPG-derived HR and HRV with ECG-derived HR and HRV.

*Given the nature of the study you may want to specify which EEG derivation were used.*
We now specified the used EEG derivations under ‘Materials’: “*Participants were equipped with eight scalp electrodes for EEG recording (providing F3/M2, F4/M1, C3/M2, C4/M1, O1/M2 and O2/M1 derivations)…*”

*I have trouble understanding the sleep onset and sleep end analysis. I usually consider SO the time in which the person falls asleep (the time of the first epoch of sleep after lights-off). Is that the case? What is the PSG event mark?*

In our study, sleep onset is the time of the first sleep epoch. Thus, the answer to your first question is yes. The PSG event mark is the time, when the participant actively presses a button on the PSG recorder. This mark indicates when the participant intends to sleep but does not sleep yet. The PSG event mark (“lights off”) allows for the calculation of additional sleep parameters such as sleep onset latency. We understand the difficulty in understanding the PSG event mark. We have elaborated the description under ‘Polysomnography’: *Participants were asked to press an event marker button on the PSG device when going to bed (identified as “lights off”). This mark represents the time when the participant intends to sleep but does not sleep yet*.

We are looking forward to your respond.

On behalf of all the authors,

Best regards,

Nanna J. Mouritzen, Medical student, Corresponding author
